# INVO Procedure: Minimally Invasive IVF as an Alternative Treatment Option for Infertile Couples

**DOI:** 10.1100/2012/571596

**Published:** 2012-05-02

**Authors:** Elkin Lucena, Angela M. Saa, Doris E. Navarro, Carlos Pulido, Oscar Lombana, Abby Moran

**Affiliations:** Centro Colombiano de Fertilidad y Esterilidad (CECOLFES) S.A.S., Calle 102 No. 14A-15, 56769 Bogotá, Colombia

## Abstract

Intravaginal culture (IVC), also called INVO (intravaginal culture of oocytes), is an assisted reproduction procedure where oocyte fertilization and early embryo development are carried out within a gas permeable air-free plastic device, placed into the maternal vaginal cavity for incubation. In the present study we assessed the outcome of the INVO procedure, using the recently designed INVOcell device, in combination with a mild ovarian stimulation protocol. A total of 125 cycles were performed. On average 6.5 oocytes per cycle were retrieved, and a mean of 4.2 were placed per INVOcell device. The cleavage rate obtained after the INVO culture was 63%. The procedure yielded 40%, 31.2%, and 24% of clinical pregnancy, live birth, and single live birth rates per cycle, respectively. Our results suggest that the INVO procedure is an effective alternative treatment option in assisted reproduction that shows comparable results to those reported for existing IVF techniques.

## 1. Introduction

Conventional* in vitro *fertilization (IVF) is the original technique of the so-called test-tube babies and currently an established treatment for infertility. The fertilization of the oocytes with the spermatozoa is performed in the laboratory, by simulating the physiological conditions to which the gametes are exposed *in vivo*. Intravaginal culture (IVC), also called INVO (intravaginal culture of oocytes), is a procedure developed by Ranoux et al., 1988, proposed as a simplified alternative option to conventional IVF [1]. In the procedure oocyte fertilization and early embryo development are carried out within a gas permeable (CO_2_ and O_2_) air-free plastic device, placed into the maternal vaginal cavity for incubation [1, 2], thus replacing the complex IVF laboratory [3]. Few years ago, INVOcell device was specially designed to overcome the difficulties and improve the results obtained with the early prototype device, which explained the occurrence of a low diffusion of the procedure for years. This new device has been ISO 10993 tested (and mouse embryos tested) to assess toxicity and biocompatibility and has received the European Union CE mark declaration of conformity [3], which is equivalent to approval by the Food and Drug Administration (FDA) in the U.S.

INVO procedure has been used worldwide and results from cycles performed by infertility centers around the world, in countries such as France, Germany, The Netherlands, England, USA, and Japan, have been published [1, 2, 4–12]. More recently our center has pioneered the use of this procedure employing the INVOcell device within the Latin-American region [13] and has been leader in its recent introduction in countries such as Mexico, Guatemala, El Salvador, Nicaragua, Dominican Republic, Panama, Venezuela, Ecuador, Peru, Bolivia, and Brazil.

In this study, we employed the INVO procedure using the INVOcell device [14], in combination with a mild ovarian stimulation protocol, with the aim of evaluating its usefulness as an alternative treatment option for infertile couples, in terms of embryonary development, clinical pregnancy, and live birth rates.

## 2. Materials and Methods

### 2.1. Setting and Design

The present study was carried out at the Colombian Center for Fertility and Sterility (CECOLFES), Bogota, Colombia. The center is certified according to ISO 9001 version 2008, and the study was approved by the institutional ethics committee, which allowed retrospective review of the patients' files.

### 2.2. Study Population

One hundred twenty (120) infertile couples with over 2 years of infertility were admitted upon giving them information and receiving their informed consent for treatment using the INVO procedure. Exclusion criteria included severe endometriosis, polycystic ovary syndrome, egg donation, and severe male factor.

From June, 2009, through May, 2011, one hundred twenty five (125) INVO cycles were performed. Population was ranked by age among four different groups to report clinical pregnancy, live birth, and single live birth rates in order to compare the outcome of the INVO procedure. Patients were distributed in groups as follows: ≤29, between 30 and 34, between 35 and 39, and ≥40 years old.

### 2.3. Controlled Ovarian Stimulation

Mild ovarian stimulation protocol was started with ovarian quiescence using oral contraceptive pills containing 150 *μ*g of desogestrel and 30 *μ*g of ethinylestradiol (Marvelon Schering Plough., Bogota, Colombia) for 3 weeks. Clomiphene citrate (50 or 100 mg; Omifin Cipla Ltd., Bernagoa, India) and human menopausal gonadotropin (one or two ampoules of 75 IU; Merional IBSA, Switzerland) were administered starting on day three of the following cycle until follicles reached 14–16 mm in diameter; at that point, blockage of follicular rupture to prevent spontaneous ovulation was achieved by administration of Indomethacin (50 mg; Genfar, Cali, Colombia) three times per day until oocyte retrieval [15]. When the dominant follicle(s) reached 17-18 mm in diameter human chorionic gonadotropin (10,000 IU; Gonacor Massone, Buenos Aires, Argentina) was injected, and 36 hours later transvaginal oocyte retrieval was performed under ultrasound guidance by traditional techniques [16].

### 2.4. Oocytes Selection

The maturity of the oocytes was morphologically assessed as traditionally, under stereo microscope with 8x magnification, based upon the expansion and radiance of the cumulus/corona oocyte complex (COC) [17], using criteria such as cumulus viscosity and dissociation of the cumulus corona radiate, additionally when possible nuclear maturity was confirmed by observation of the polar body.

### 2.5. Intravaginal Culture Procedure

**Figure 1:**
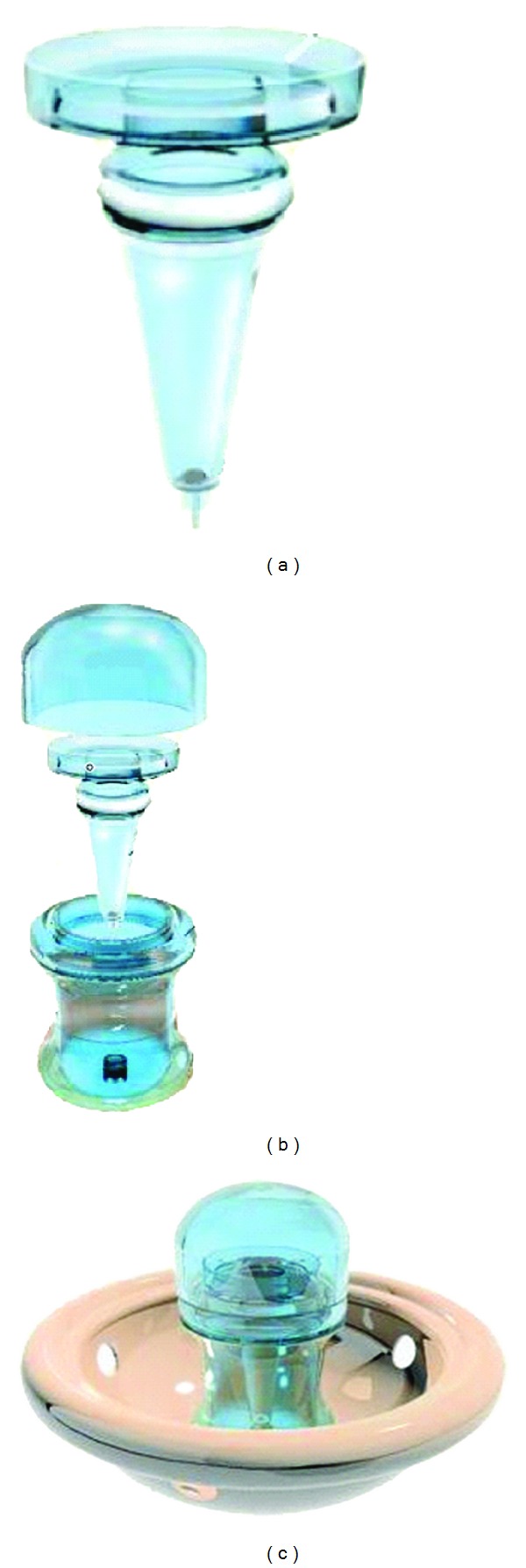
Component parts of the INVOcell device. (a) Inner chamber. (b) Outer rigid shell. (c) Assembled INVOcell device with its retention system.

The INVOcell device (INVO Bioscience., Beverly, MA, USA, http://www.invobioscience.com/) is composed of an inner chamber with a rotating valve and a protective outer rigid shell (Figure 1). The INVO procedure was performed as previously described with some modifications [3]. Semen samples were treated by the *swim-up* method [18]. Initially the device inner chamber is filled with pregazed and prewarmed G2 Plus version 5 medium (Vitrolife AB, Goteborg, Sweden), then a count of 35.000–50.000 spermatozoa are loaded, followed by the selected number of oocytes (4.2 in average). After assembly of the device, it is immediately positioned into the vaginal cavity, in proximity to the uterine cervix, altogether with a diaphragm as retention system. Some recommendations were given to patients to follow during the culture period such as restraining from intercourse and abstention of tub bathing, douching, or swimming.

After a 72-hour culture period the INVOcell device was removed, embryos were retrieved and immediately evaluated according to their development and fragmentation degree. The selected embryos were transferred under transabdominal ultrasound guidance.

### 2.6. Luteal Phase Support and Pregnancy Determination

Estradiol Valerianate (4 mg; Delpharm Lille SAS, Paris, France) and natural oily progesterone (100 mg; Ryan Laboratories, Bogota, Colombia) were given daily during 6 days starting the day of transfer, followed by vaginal progesterone (600 mg/day; Utrogestan Besins International, Paris, France) until day 12 after-transfer, when serum **β*-*HCG pregnancy determination was performed. Seven weeks after transfer, the presence of gestational sac with fetal heart beat by ultrasonography was used to confirm the clinical pregnancy.

### 2.7. Vitrification

In some patients, supernumerary oocytes confirmed to be mature by first polar body observation after denudation, as well as those good quality embryos that were not transferred, were vitrified for use in subsequent cycles. Vitrification was performed according to the protocol previously published by our group [19].

### 2.8. Outcome Measures and Statistical Analysis

Primary outcome measures included pregnancy, live birth, and single live birth rates per transfer. Secondary outcome measures included mean numbers of retrieved oocytes and oocytes placed per INVO device, as well as embryo cleavage and transfer rates after INVO culture. Statistical analysis, to compare our results with published data concerning conventional IVF outcomes, was performed using Student's *t*-test. *P* < 0.05 was considered statistically significant.

## 3. Results

One hundred twenty-five (125) cycles combining the INVO procedure and mild ovarian stimulation protocol were performed. There was no cycle cancellation, as all started cycles went to retrieval. A total of 812 oocytes were retrieved, for an average of 6.5 per puncture. A mean of 4.2 oocytes were placed for insemination per the INVO device. On average 2.6 embryos per cycle were obtained, for a cleavage rate of 63%, out of which a mean of 2.1 embryos were transferred per cycle, for a total of 114 transfers (91.2%). Cycle distribution per patient's age groups was seventeen for ≤29, 54 for 30–34, 48 for 35–39, and 6 for ≥40 years old (Table 1).

### 3.1. Patients ≤ 29 Years Old

A total of 128 oocytes were retrieved (mean value 7.5), out of which 78 were selected for insemination (average 4.6). After intravaginal culture, 46 embryos (on average 2.7 per cycle) were obtained, out of which an average of 2.3 embryos per cycle were selected for transference, and the remaining viable embryos were cryopreserved. Twelve positive *β*-HCG tests were obtained, out of which 10 clinical pregnancies were confirmed (Table 1). Up to date 6 pregnancies have successfully reached live birth with eight healthy children born, and there is one ongoing pregnancy.

### 3.2. Patients between 30 and 34 Years Old

A total of 352 oocytes were retrieved (mean value 6.5), out of which 207 were selected for insemination (average 3.8). After intravaginal culture, 152 embryos (on average 2.8 per cycle) were obtained, out of which an average of 2.3 embryos per cycle were selected for transference, and the remaining viable embryos were cryopreserved. Twenty-six positive *β*-HCG tests were obtained, out of which 22 clinical pregnancies were confirmed (Table 1). Up to date 12 pregnancies have successfully reached live birth and 17 healthy children were born, and there are 5 ongoing pregnancies.

### 3.3. Patients between 35 and 39 Years Old

A total of 229 oocytes were retrieved (mean value 6.2), out of which 205 were selected for insemination (average 4.3). After intravaginal culture, 121 embryos (on average 2.5 per cycle) were obtained, out of which an average of 2.0 embryos per cycle were selected for transference, and the remaining viable embryos were cryopreserved. Eighteen positive *β*-HCG tests were obtained, out of which 17 clinical pregnancies were confirmed (Table 1). Up to date 12 pregnancies have successfully reached live birth and 17 healthy children were born, and there are 2 ongoing pregnancies.

### 3.4. Patients ≥ 40 Years Old

A total of 33 oocytes were retrieved (mean value 5.5), out of which 30 were selected for insemination (average 5.0). After intravaginal culture, 9 embryos (on average 1.5 per cycle) were obtained, out of which an average of 1.5 embryos per cycle were selected for transference, and the remaining viable embryos were cryopreserved. One clinical pregnancy was confirmed (Table 1), up to date this is a healthy ongoing pregnancy.

 Out of the 125 cycles, 11 were not transferred and in two of them there were well-developed embryos but transfer could not be achieved, one because an abnormal uterine bleeding and the other due to the presence of bicornuate uterus in retroversion, in both cases embryos were vitrified. In the remaining 9 cycles there was either not fertilization (seven) or an inappropriate embryo development (two). None of the 120 patients reported any discomfort while using the INVOcell device or presented signs of infection.

## 4. Discussion

In the present study we assessed the outcome of the INVO procedure, using the specially designed INVOcell device, in terms of pregnancy, live birth, and single live birth rates; our results showed comparable successful rates with traditional IVF, highlighting its usefulness as an alternative option treatment in assisted reproduction. 

**Figure 2:**
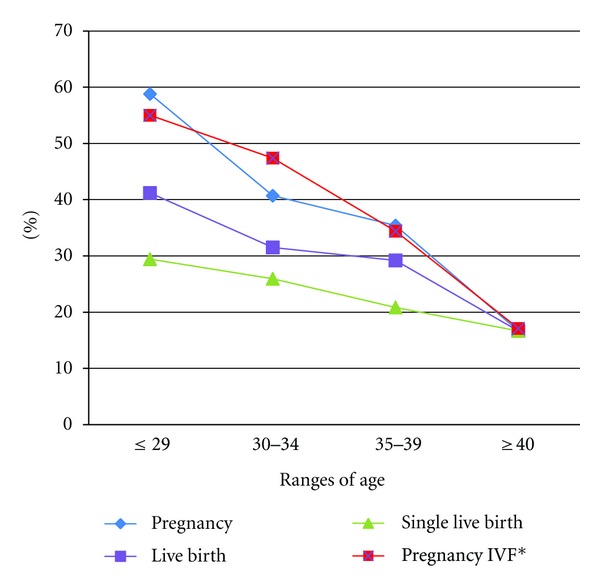
Rates of primary outcomes: pregnancy, live birth, and singleton live birth from INVO procedure. *From the US Centers for Disease Control and Prevention (CDC), 2009 National Summary Report. *P* > 0.05 for pregnancy rates between traditional IVF versus INVO (*T*-test). For INVO results, live birth and single live birth rates include up to date ongoing pregnancies.

The US Centers for Disease Control and Prevention (CDC) provides cumulative summary statistics on the outcomes of IVF cycles; the most recent complete data available are from 2008 [20]. During that year the pregnancy, live birth, and singleton live birth rates per oocyte retrieval were 41.6%, 33.8%, and 23%, respectively. These results are comparable to ours from INVO cycles as we obtained 40%, 31.2%, and 24%, respectively, for these outcomes. Patients' age was the most predictive factor of success with a marked decrease across the groups of age from ≤29 until ≥40 years old, in terms of pregnancy, live birth, and single live birth rates (Figure 2). More recently available data report a pregnancy rate per cycle of 55% for patients of 29 years old and under, 47% for patients between 30 and 34, 34% for patients between 35 and 39, and 17% for patients 40 years old and older [21]. These results are also comparable to ours for the INVO procedure, where as expected pregnancy rates decreased with maternal age, from 58.8%, through 40.7% and 35.4%, until 16.7%, for woman aged ≤ 30, 30–34, 34–39, and ≥ 40 years old, respectively. There were no statistical differences for pregnancy rate through age groups between the reported data and our results (*P* = 0.96) (Figure 2). Taken together, these results suggest that the INVO procedure could be an alternative treatment for infertile patients ensuring success rates comparable to those in the existing IVF techniques.

Cycle secondary outcomes, including mean numbers of retrieved oocytes and oocytes placed per INVO device, in addition to embryos cleavage and transfer rates after INVO culture, were comparable between the first three established groups of age. However, the last group of age (≥40 years old) showed notably lower mean values within these measures (Table 1).

The higher incidence of multiple pregnancies secondary to IVF is well recognized. The 2008 CDC annual report indicates a 55.5% of singleton and 26% of multiple live births (20). In comparison, in our results the multiple gestation rate was 1.4 times less (18%) than the reported. Our pregnancies were distributed as follows: 60% single, 12% twins, and 6% triple live births, as a result of transferring on average 2.4 embryos per transferred cycle. The advances of ART have involved among other things evolution of culture media components; however, the environmental conditions influence drastically the embryo quality by their effect on O_2_ and CO_2_ concentration, pH, and temperature [22–25]; a misbalance in this components may induce oxidative stress, responsible for inappropriate early development and embryo fragmentation [26]. Oxygen is an important player during embryo development [27]; the INVO procedure offers an *in vivo* fertilization environment with the effectiveness of an oxygen concentration that closely resembles the uterine cavity atmosphere of less than 5% of oxygen. This concentration of oxygen ensures the energetic metabolism required for a successful gametes viability, activation, fertilization, and embryo development, which takes place under near anaerobic conditions [28]. On the other hand, CO_2_ is a key atmospheric component during embryo development; in the culture media it produces carbonic acid that is equilibrated with sodium bicarbonate originating the optimal pH necessary for embryo development [29]. In traditional large gas-filled incubators, the CO_2_ concentrations may vary because of repeated opening, impacting the pH equilibrium in the culture medium, decreasing embryo quality [30]. In contrast the INVOcell device is a closed system able to keep temperature and pH stability during 72 h of uninterrupted embryo culture, providing a stable, pure, inexpensive, and easy-access source of the required oxygen/CO_2_ concentration. A limitation of the intravaginal culture would be the inability to monitor or adjust gas levels; however, according to our results we could ensure that this system is capable of maintaining an environmental stability that achieves comparable results to those obtained with large gas-filled incubators [29, 31]. In our study, from 520 oocytes loaded into the INVOcell device, 326 viable embryos (with a proper development of 6–8 cells after 72 hours of culture) were retrieved; additionally 79.6% of these embryos were free of fragmentation. These results suggest that the INVOcell procedure offers an appropriate embryo development with an optimal quality for implantation success. Infertile couples during assisted reproduction treatments undergo an intense process that involves physiological difficulties, physical discomfort, psychological and economical implications, in addition to the medical procedures that represent risks and secondary effects [32, 33]. A significant aspect of the INVO procedure lies in the psychological benefit that is created among the patients who feel closely involved in the process of fertilization and early embryo development, what generates a high level of acceptance of the procedure. The INVOcell is placed into the vaginal cavity altogether with a retention system (diaphragm) (Figure 1(c)) that situates it close to the uterus, the holes in the diaphragm membrane avoid the accumulation of vaginal secretions that would be responsible for infection induction during intravaginal culture period. Accordingly in our study no patient reported any infection or physical discomfort while or after using the device.

An advantage of the INVO procedure is the fact that by simplifying the laboratory equipment and manipulation needed, it might decrease the costs and allow a widespread application for patients who cannot afford traditional IVF [34], as it would be the case in developing countries, where the access to cost-effective infertility treatments is limited.

This study suggests that intravaginal culture using the INVOcell device could be a viable alternative option for assisted reproduction. However, additional prospective and probably multicentric studies, involving higher number of cycles, would be necessary to confirm its efficacy and safety. Additionally it would be interesting to investigate the outcome and usefulness of the INVO culture system in conjunction with the ICSI technique, to evaluate its applicability for indications such as severe male factor.

## Figures and Tables

**Table 1:** Summary of results from the INVO procedure.

Groups^a^	Cycles (*n*)	Transfers^b^ (*n*)	Retrieval^c^	INVOcell^d^	Cleavage^e^	ET^f^	Pregnancy^g^
≤29	17	16 (94,1)	7,53	4,59	2,7 (58,7)	2.3	10 (58,8)
30–34	54	51 (94,4)	6,52	3,8	2,8 (73,7)	2.3	22 (40,7)
35–39	48	43 (89,6)	6,23	4,27	2,5 (58,5)	2.0	17 (35,4)
≥40	6	4 (66,7)	5,5	5	1,5 (30)	1.5	1 (16,66)

Total	125	114 (91,2)	6,5	4,2	2,6 (63)	2,1	50 (40)

INVO: intravaginal culture of oocytes; ET: embryo transfer. Values in parentheses are percentages.

^a^Ranges of ages. ^b^Cycles that ended up in transfer. ^c^Mean number of retrieved oocytes per punction. ^d^Mean number of oocytes placed for fertilization per INVOcell device (cycle). ^e^Mean number of retrieved viable proper developed embryos per INVOcell device (cycle). ^f^Mean number of transferred embryos per cycle. ^g^Number of clinical pregnancies per cycle.
